# Higher visual responses in the temporal cortex of mice

**DOI:** 10.1038/s41598-018-29530-3

**Published:** 2018-07-24

**Authors:** Nana Nishio, Hiroaki Tsukano, Ryuichi Hishida, Manabu Abe, Junichi Nakai, Meiko Kawamura, Atsushi Aiba, Kenji Sakimura, Katsuei Shibuki

**Affiliations:** 1 Department of Neurophysiology, Brain Research Institute, Niigata University, Niigata, 951-8585 Japan; 2 Department of Cellular Neurobiology, Brain Research Institute, Niigata University, Niigata, 951-8585 Japan; 3 Graduate School of Science and Engineering, Saitama University, Saitama, 338-8570 Japan; 4 Brain and Body System Science Institute, Saitama University, Saitama, 338-8570 Japan; 5 Laboratory of Animal Resources, Center for Disease Biology and Integrative Medicine, Faculty of Medicine, The University of Tokyo, Tokyo, 113-0033 Japan

## Abstract

The visual cortex of mice is a useful model for investigating the mammalian visual system. In primates, higher visual areas are classified into two parts, the dorsal stream (“where” pathway) and ventral stream (“what” pathway). The ventral stream is known to include a part of the temporal cortex. In mice, however, some cortical areas adjacent to the primary visual area (V1) in the occipital cortex are thought to be comparable to the ventral stream in primates, although the whole picture of the mouse ventral stream has never been elucidated. We performed wide-field Ca^2+^ imaging in awake mice to investigate visual responses in the mouse temporal cortex, and found that the postrhinal cortex (POR), posterior to the auditory cortex (AC), and the ectorhinal and temporal association cortices (ECT), ventral to the AC, showed clear visual responses to moving visual objects. The retinotopic maps in the POR and ECT were not clearly observed, and the amplitudes of the visual responses in the POR and ECT were less sensitive to the size of the objects, compared to visual responses in the V1. In the ECT, objects of different sizes activated different subareas. These findings strongly suggest that the mouse ventral stream extends to the ECT ventral to the AC, and that it has characteristic response properties that are markedly different from the response properties in the V1.

## Introduction

In primates, visual information is processed by two main visual pathways originating from the V1, the dorsal and ventral streams^1–4^. The dorsal stream (“where” pathway) is specialized for analyzing movements and spatial information of visual stimuli, whereas the ventral stream (“what” pathway) is important for processing shape and texture of visual stimuli. The what pathway extends to the temporal cortex, and contains a group of neurons responding to a specific category of visual objects^5–12^. In the rodent visual system, the V1 has been extensively studied and properties of some higher visual cortices connected with the V1 have been elucidated by histological^13,14^ and functional studies^15–23^. These studies suggest that, in rodents, the medial and anterior parts of the occipital cortex around the V1 correspond to the dorsal pathway, and the lateral part belongs to the ventral pathway. In rats, neurons located in the lateral parts of higher cortices are less affected by the position or brightness of a stimulus, and this property is one of the characters expected in the neurons of the ventral stream^22,23^. However, previous studies using rodents have not elucidated the whole picture of the ventral stream, and it is not clear whether more lateral or ventral parts, including a part of the temporal cortex, can show higher visual responses when stimulated properly.

The skull of the mouse is transparent, and transcranial imaging of cortical activities is therefore possible. We investigated cortical activities in the mouse using this method, based on activity-dependent endogenous fluorescence signals derived from mitochondrial flavoproteins^24^. Unfortunately, the intensity of the endogenous fluorescence is weak and strongly affected by activity-dependent hemodynamic responses^25^, especially in unanesthetized mice. Since neural responses in the higher visual cortex might be affected by anesthesia^26^, it may be appropriate to investigate the properties of the murine higher visual cortex in unanesthetized animals. Recently, various types of Ca^2+^-sensitive fluorescent proteins have been developed^27,28^. Since the intense fluorescence derived from G-CaMP8, a Ca^2+^-sensitive fluorescent protein^29^, is less affected by hemodynamic responses compared to the weak flavoprotein fluorescence, we investigated visual responses in awake mice that express G-CaMP8 in cortical excitatory neurons. We especially focused on the postrhinal cortex (POR), one of the putative higher visual areas in the murine ventral stream, since the POR locates most ventrally among the known visual cortices^17,19,30^. We identified the POR based on the retinotopic map reported previously. We also focused on the ectorhinal cortex and the adjacent temporal association cortex (taken together, referred to as ECT), which is anatomically connected with the POR and locating ventral to the auditory cortex^14^. We found that the POR and the cortical area corresponding to the ECT exhibit marked visual responses to moving objects, and that their response properties are quite different from those in the V1. These findings strongly suggest that the higher visual responses in the POR and ECT of mice may be counterparts of higher responses in the ventral stream of primates.

## Results

### Responses to moving disc stimulation

**Figure 1:**
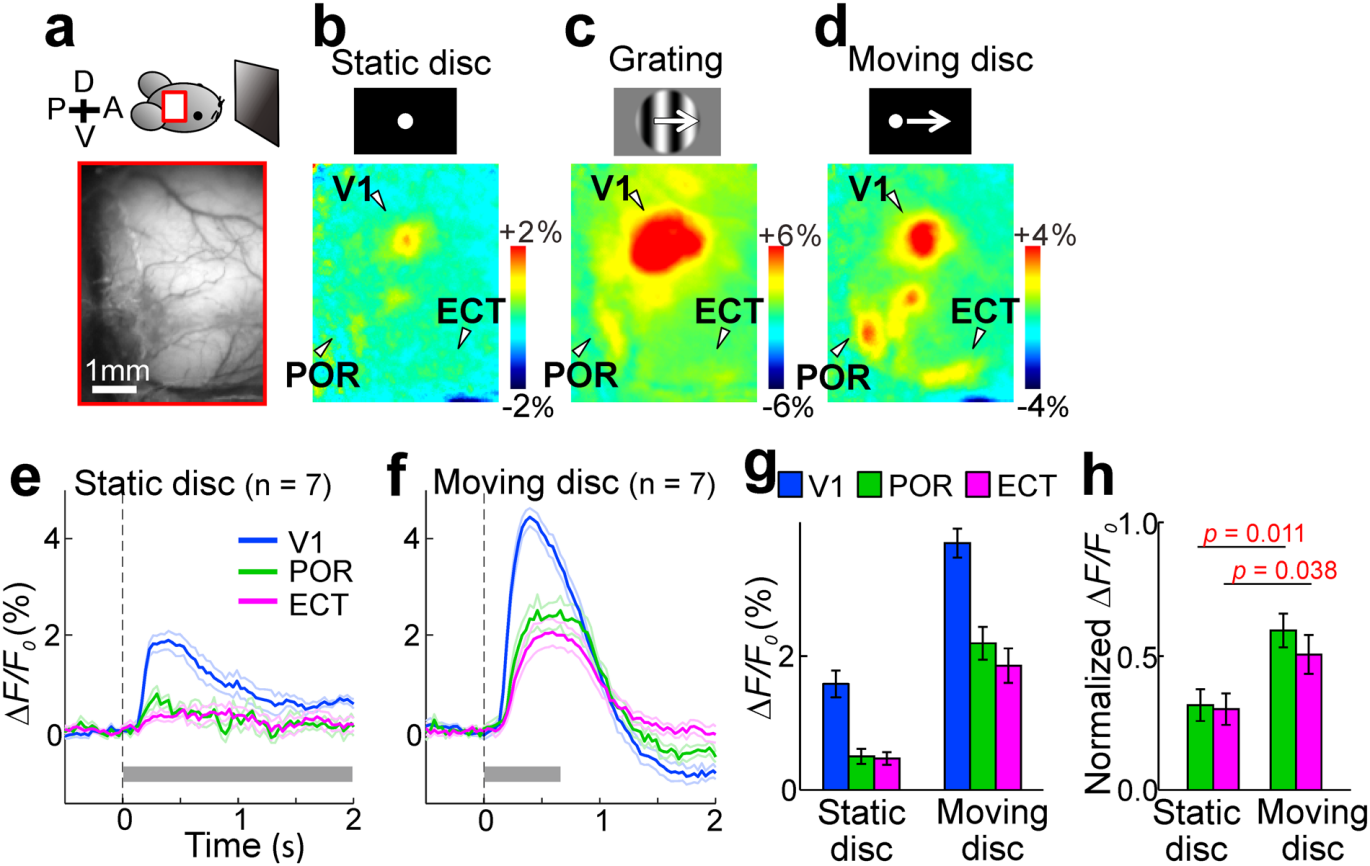
Visual responses in the temporal cortex. (**a**) Experimental setup. A monitor for visual stimuli was placed in front of a mouse. An imaging area is schematically indicated with a red square. Vascular images of the cortical surface of the imaged area are also shown. A: anterior; P: posterior; V: ventral; D: dorsal. (**b–d**) Visual responses to a static disc (diameter: 5°, (**b**) drift grating patterns (50°, **c**), or a moving disc (5°, **d**) in the same mouse as shown in (**a**). (**e**,**f**) Changes in ΔF/F_0_ recorded in the V1 (blue), POR (green), and ECT (magenta) in responses to visual stimuli presented during the period indicated by gray bars. Mean and SEM (thin lines) are shown. Seven mice were used. Circular regions of interests (ROIs, diameter: 0.3 mm or nine pixels) placed at the response peak in the V1, POR and ECT were used to measure response amplitudes. (**g**) Response amplitudes to visual stimuli in the same mice shown in (**e**,**f**). A two-way ANOVA showed significant effects of cortical area (p < 0.0001) and stimulus type (p < 0.0001), but not in interaction (p = 0.207). (**h**) Response amplitudes in ΔF/F_0_ normalized by those of V1 responses. The same data shown in (**g**). Statistical significance was evaluated using the Mann-Whitney U test. Red values: p < 0.05.

**Figure 2:**
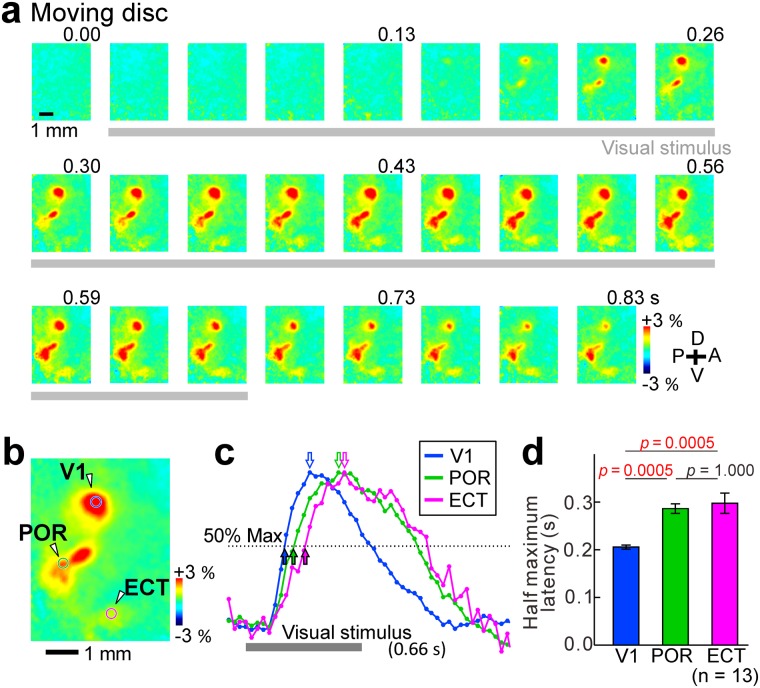
Latency of visual responses in the V1, POR and ECT. (**a**) Sequential images of the visual responses to a moving disc. Gray bars show the timing of visual stimulation. Numbers show the time after stimulus onset. (**b**) Averaged visual responses from 0.2 to 0.5 s after stimulus onset in (**a**). (**c**) Normalized fluorescence changes in the V1 (blue), POR (green) and ECT (magenta). ROIs are shown in (**b**). Open arrows: timing at the maximal responses (100%). Filled arrows: timing at 50% of the maximal responses. (**d**) Half maximum latency after stimulus onset in the V1, POR and ECT. Statistical significance was evaluated by the Kruskal-Wallis test followed by the Dunn-Sidak test. Red values: p < 0.05.

Visual stimuli were presented in front of the mouse and responses were imaged in a wide area including the V1 and the auditory cortex. When stimulated with the presentation of a static disc or drifting grating patterns, V1 was mainly activated (Fig. 1a–c). Various higher visual areas surrounding the V1 were also activated, as reported previously^19,20^. We focused on the POR that locates most ventrally among the visually responsive areas with a retinotopic map (see later results). We were also interested in the ectorhinal cortex with surrounding and temporal association areas (ECT) that has been reported to receive projections from the POR^14^. We expected that the ECT was in a ventral part of the temporal cortex just dorsal to the rhinal fissure. The activities in the POR were very weak and almost no response was found in the area corresponding to the ECT, compared with the responses in the V1. The POR was more intensely activated by drifting grating patterns than by a static object, and we therefore speculated that movement of visual stimuli could be an important factor for stimulating the POR. As expected, the POR was markedly activated by a moving disc at a speed of 60°/s (Fig. 1d). Furthermore, the cortical activities were clearly observed in ventral parts of the temporal association cortex including the ECT (Fig. 1d). However, V1 responses to a moving disc were also increased compared with those to a static disc (Fig. 1e–g). A two-way ANOVA showed significant effects of cortical area (p < 0.0001) and stimulus type (p < 0.0001), but not in interaction (p = 0.207). Then, we normalized response amplitudes in the POR and ECT by those in the V1. Normalized responses to a moving disc were significantly larger than those to a static disc in the POR and ECT (Fig. 1h). As the ECT is located near the auditory cortex, we compared the visual responses to a moving disc and the auditory responses to tonal stimuli and confirmed that the visual responses appeared in the ECT ventral to the auditory cortex (Fig. S1)^31,32^. The visual responses to a moving disc appeared first in the V1, and then in the POR and ECT, in this order (Fig. 2a), indicating that the POR and ECT are higher areas that receive visual information from the V1. We measured the half maximum latency (Fig. 2b–d), because this parameter was less sensitive to signal drifting in our optical recordings. Our results are larger than the previously reported values measured as the time at which the response became 2 SD as large as background signals derived from a voltage-sensitive dye^33^. The 2 SD latency in our data was shorter than the half maximum latency (Fig. S2), but still larger than the previous results^33^, possibly due to the differences between calcium and voltage signals.

### Direction and speed preferences of higher visual responses

**Figure 3:**
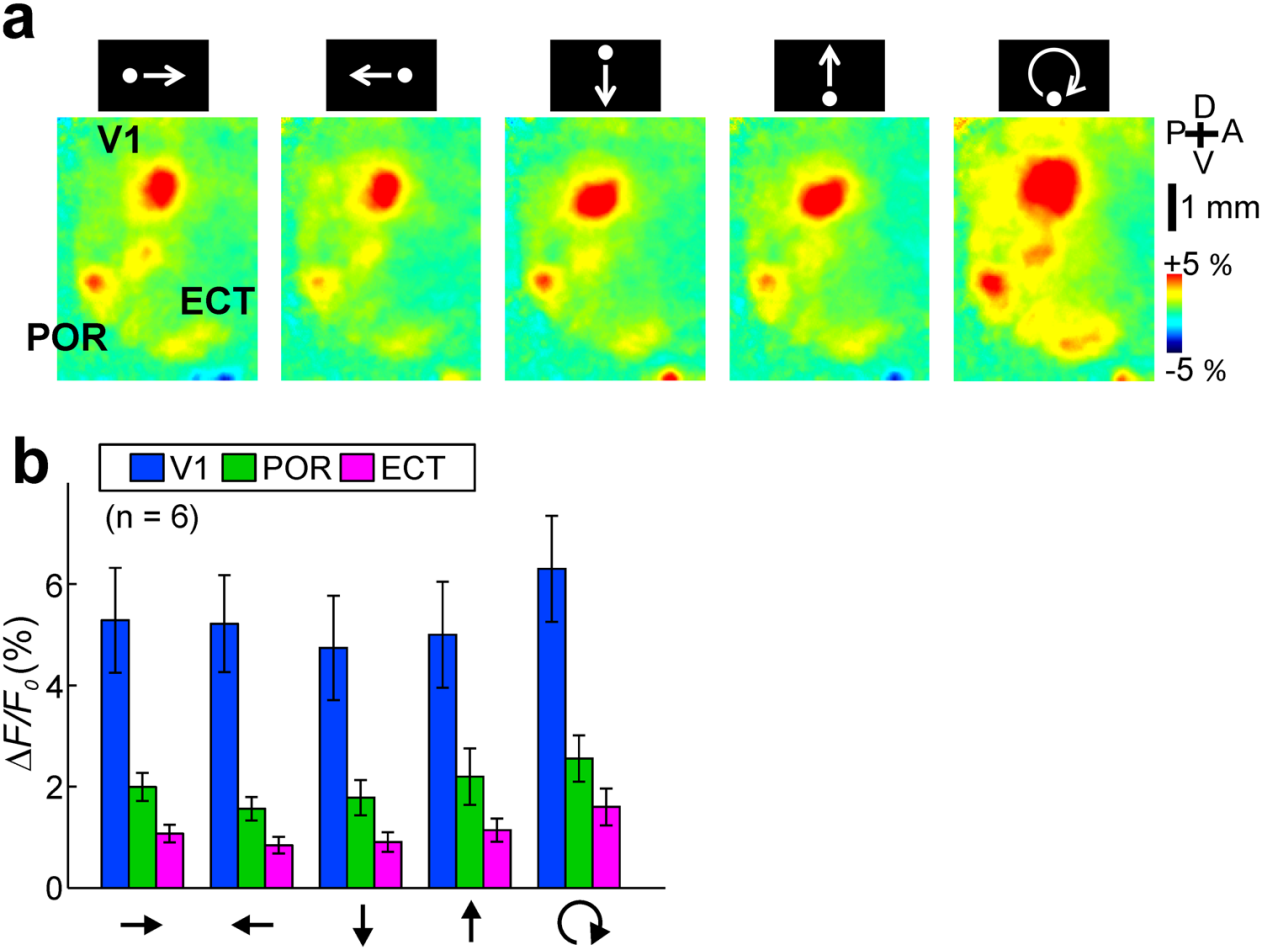
Direction preferences in the V1, POR and ECT. (**a**) Visual responses to a moving disc to rightward (leftmost), leftward (middle left), downward (middle), upward (middle right), and rotating (rightmost) movements. (**b**) Response amplitudes to each stimulus in the V1 (blue), POR (green) and ECT (magenta). A two-way ANOVA showed no significant effects of stimulus type (p = 0.339) and of interaction with cortical area (p = 0.997), and a significant effect of cortical area type (p < 0.0001).

Since the movement of stimuli was found to be important for eliciting neural responses in the POR and ECT, we further investigated whether any direction selectivity was observed in the responses. Visual responses were similarly induced by stimuli with rightward, leftward, downward, upward, and rotational movements in the visual field (Fig. 3). A two-way ANOVA test showed a significant effect of cortical area type (p < 0.0001), but no significant effects of stimulus type (p = 0.339) and of interaction with cortical area (p = 0.997). The lack of direction tuning was expected from the salt-and-pepper organization of neuronal tuning in mice and the fact that our imaging approach was not able to resolve the activity of single cells. As a matter of fact, previous studies have shown that at least a fraction of the neurons in V1 and extra striate visual cortex in mice are direction selective^15,17,34^.

**Figure 4:**
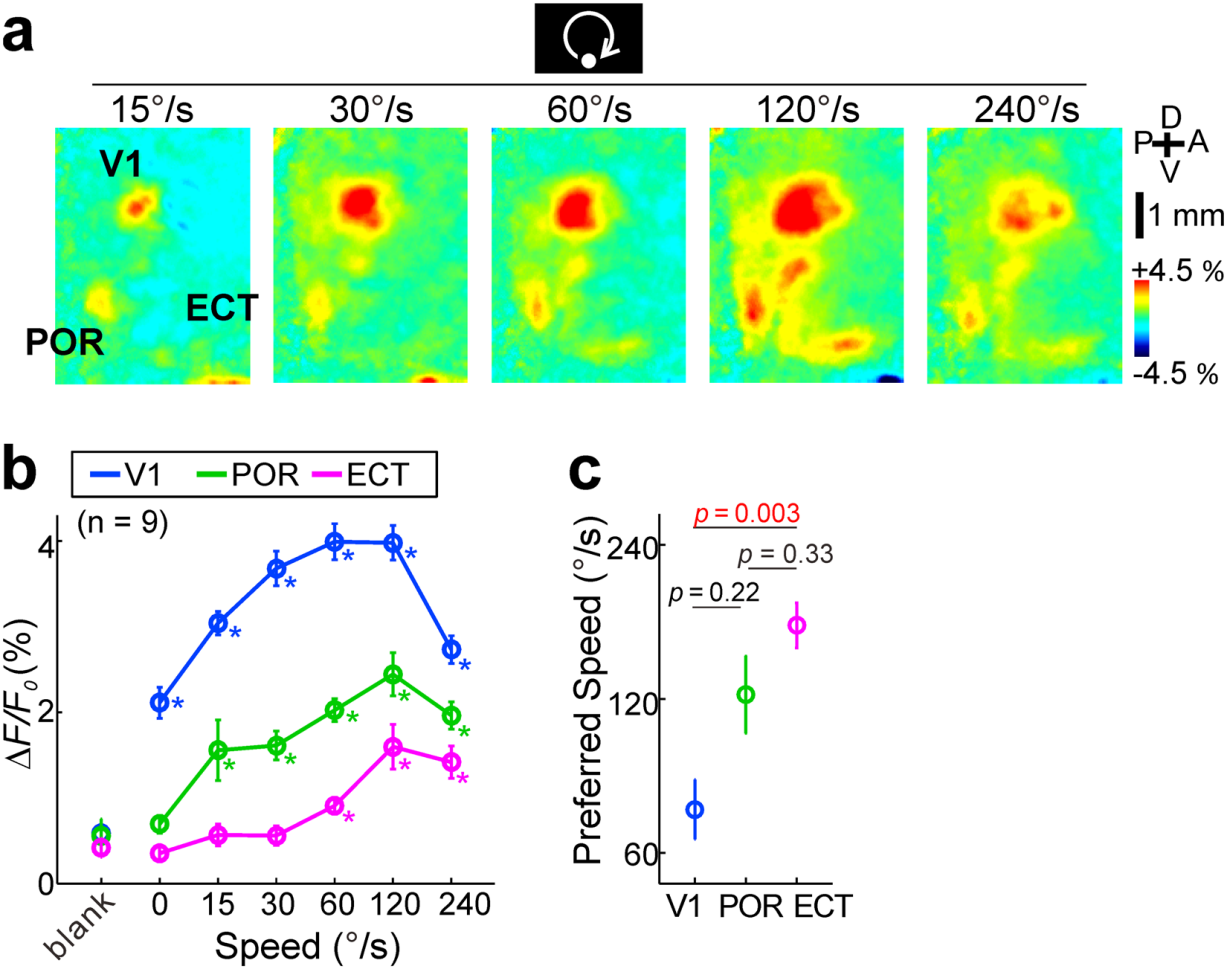
Speed preferences in the V1, POR and ECT. (**a**) Visual responses to a moving disc with speed 15, 30, 60, 120 or 240°/s. (**b**) Response amplitudes and speeds of rotating disc stimulation or the absence of stimulation (blank) in the V1 (blue), POR (green) and ECT (magenta). A two-way ANOVA detected significant effects of stimulus type, cortical area type and their interaction (p < 0.0001, each). Asterisks: p < 0.05; the Mann-Whitney U test with Holm’s correction for multiple comparisons with compared to each blank, n = 9. (**c**) Preferred speeds estimated from the peak of the fitted Gaussian in the V1 (n = 9), POR (n = 8) and ECT (n = 7). Statistical significance was evaluated by with the Kruskal-Wallis test followed by the Dunn-Sidak test. Red values: p < 0.05.

Using rotationally moving discs, we investigated the dependence of visual responses on the stimulus speed (Fig. 4), and found that responses were observed at the speed ≥0°/s in the V1, ≥15°/s in the POR, ≥60°/s in the ECT (Fig. 4a). A two-way ANOVA test showed significant effects of cortical area type, stimulus type and their interaction (p < 0.0001 each, Fig. 4b). We mainly used a speed of 60°/s in the following experiments, since we could observe clear responses in the V1, POR and ECT at this speed. However, we thought that the speed preferences should be investigated in detail for comparing with previous studies^15,23^. Speed preferences in the three areas were compared by measuring the peak position of a Gaussian curve that fitted to response amplitudes (Fig. 4b,c). The estimated preferred speed tended to be faster in the order of V1, POR and ECT, and it turned out that the speed of V1 and ECT was significantly different (Fig. 4c). These results are consistent with previous study that neurons in the rat temporal visual cortex are more responsive to moving stimuli than static stimuli^23^.

### Retinotopic maps

**Figure 5:**
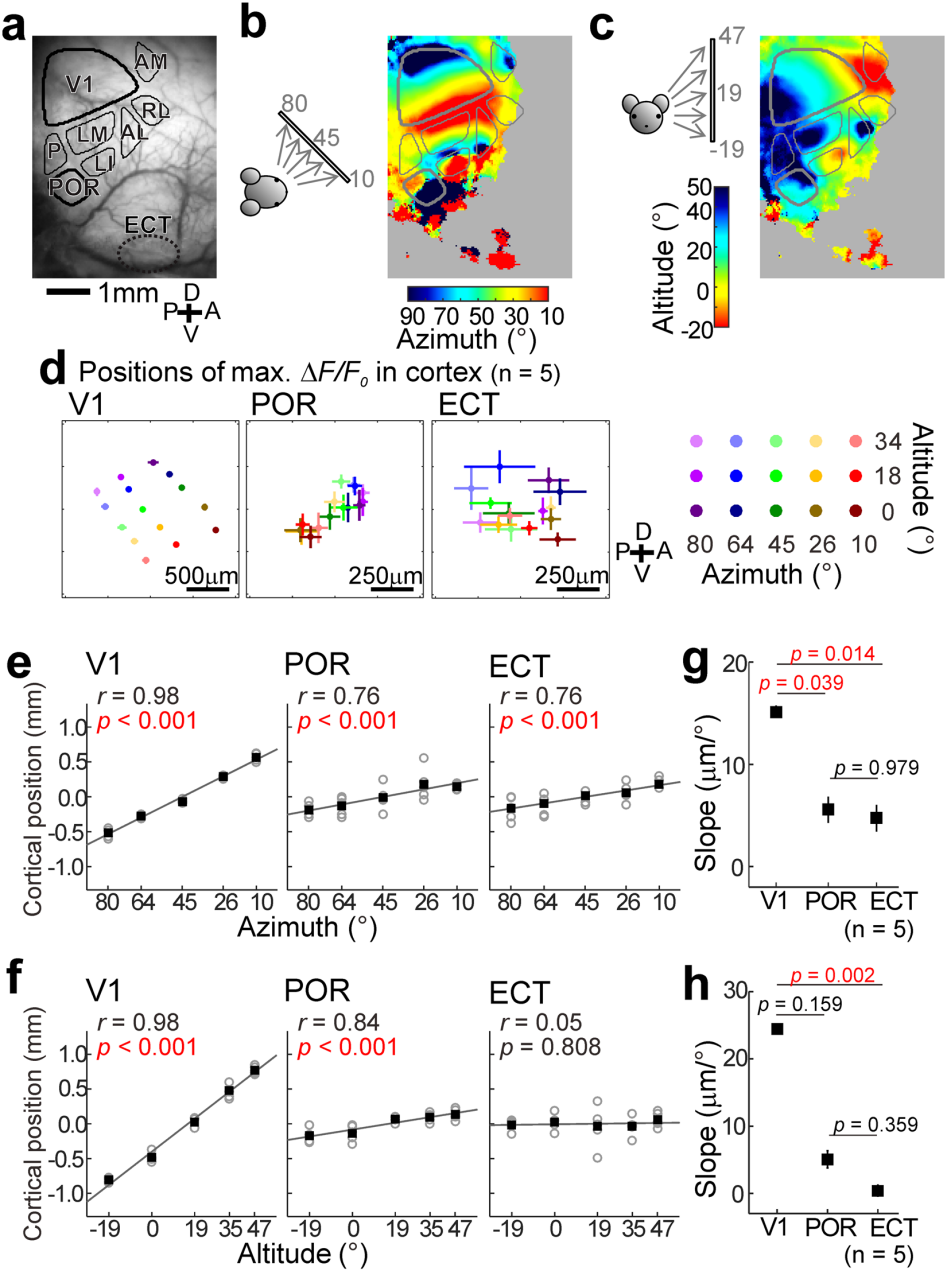
Retinotopic maps. (**a**) Cortical images with identified visual cortical areas. (**b**,**c**) Retinotopic maps to horizontal (**b**) and vertical (**c**) positions of a rotating disc in the visual field. Only pixels with ΔF/F_0_ > 2% are colored. (**d**) Positional plots of maximal ΔF/F_0_ in the V1 (left), POR (middle left) and ECT (middle right) in a rotating disc located at various sites in the visual field (right). Results obtained from five mice were used to estimate the mean and SEM of maximal ΔF/F_0_ position. The mean of the coordinates of maximal ΔF/F_0_ position was used as the origin of the coordinates to superimpose the data in five mice. (**e**,**f**) Correlation between stimulus position in the horizontal (**e**) or vertical (**f**) visual field and cortical position of maximal ΔF/F_0_ in the V1 (left), POR (middle) and ECT (right). Open circles: data in each mouse; filled squares: mean. Plots were obtained from the responses to the stimuli presented at 0° in altitude (**e**) or 27° in azimuth (**f**). Cortical position (vertical axis) was measured along the approximate straight lines connecting the response areas. Statistical significance of correlation was estimated using the Spearman rank correlation test. (**g**,**h**) Comparison of slopes of approximate straight lines in (**e**,**f**) between the V1, POR and ECT. The Kruskal-Wallis test followed by the Dunn-Sidak test were used for estimating p values. Red values: p < 0.05 (n = 5).

In primates, the receptive field of higher visual neurons in the cortex is large, and the retinotopic maps become less clear in higher areas^35,36^. We tested whether this property of retinotopic maps is also found in mice. In this experiment, stimulus position using a moving disc must be localized to obtain retinotopic maps. Therefore, we used a rotationally moving disc, which satisfies this requirement. The optimal positions (azimuth and altitude) of the stimuli in each pixel in the cortical image were estimated from the peak of the Gaussian curves that fitted to response amplitudes, and retinotopic maps were constructed from these data (Fig. 5a–c). A clear map was observed in the V1 but not in the POR, as reported previously^17,19^. Furthermore, no clear map was found in the ECT. To investigate the properties of retinotopic maps, we determined the position of maximal ΔF/F_0_ in the V1, POR and ECT (Fig. 5d), and the relationship between the stimulus position and the location of maximal ΔF/F_0_ was analyzed in the azimuth (Fig. 5e,g) and altitude (Fig. 5f,h). For comparing the results between the V1, POR and ECT, we measured the slope of the cortical distance between the points of maximal ΔF/F_0_ divided by the distance in the azimuth (Fig. 5g) and altitudes (Fig. 5h) of the visual field. The slopes in the ECT was significantly lower than those in the V1 (Fig. 5g,h), indicating that the retinotopic map in the ECT was ambiguous compared with that in the V1. When this analysis was performed after taking into account the different size of the visual areas (i.e., by using normalized cortical distances), we found a significantly smaller slope in ECT than in V1 in the altitude (Fig. S3). These results indicate that the ambiguous retinotopic maps in higher visual areas, which have been found in primates, were also observed in mice.

### Preference of stimulus size in higher visual areas

**Figure 6:**
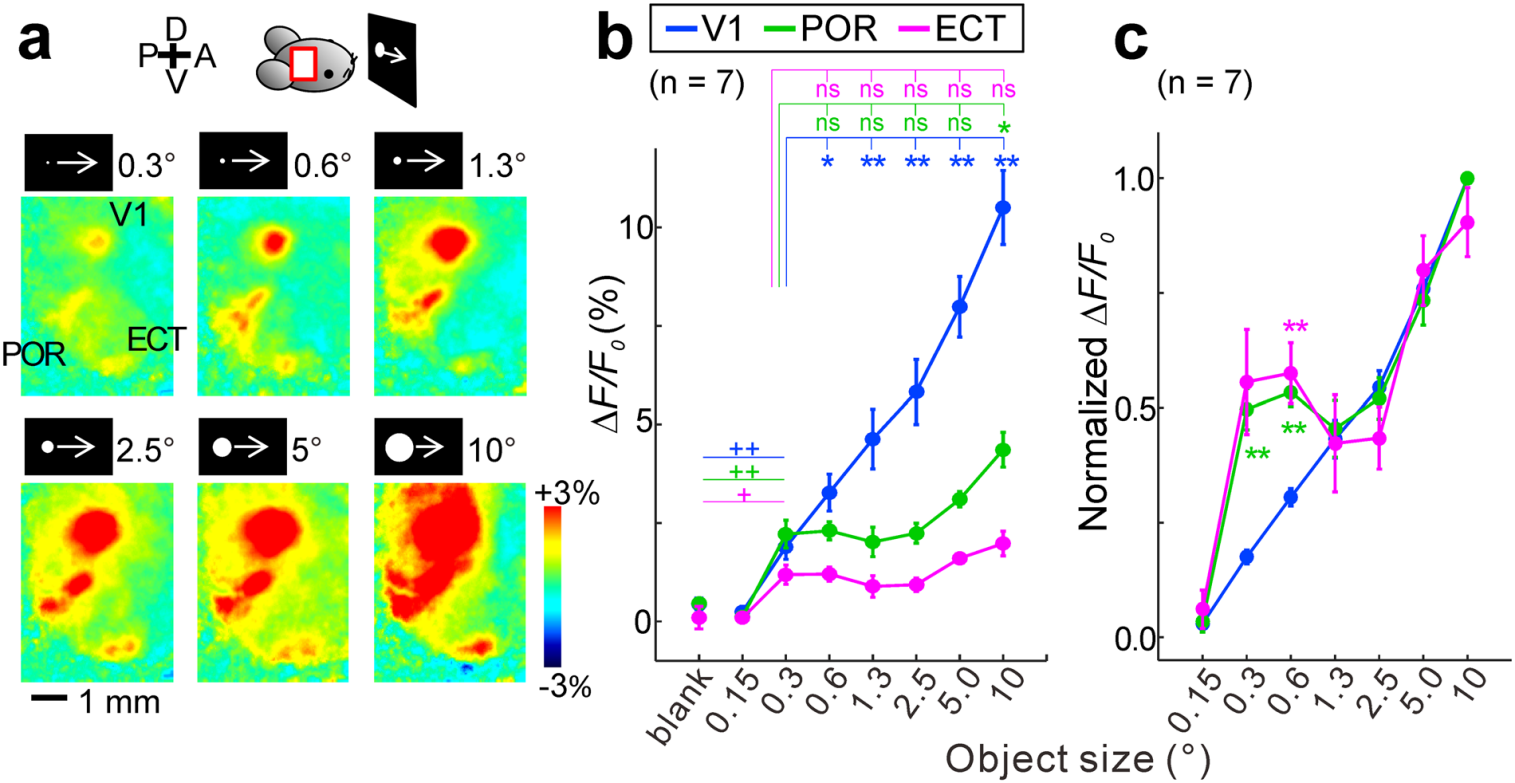
Response amplitudes to bright moving discs of various sizes. (**a**) Responses to bright moving discs with a diameter between 0.3° and 10°. (**b**) Response amplitudes and diameters of the bright moving discs. Circular ROIs (diameter: 0.3 mm or nine pixels) were used to measure the amplitudes. A two-way ANOVA showed significant effects of stimulus type, cortical area type and their interaction (p < 0.0001). Significant responses were found in response to a moving disc at 0.3° (^++^p < 0.01, ^+^p < 0.05, the Mann-Whitney U test with Holm’s correction). No significant difference was found between 0.3° and 5° within the POR and ECT (**p < 0.01, *p < 0.05, the Mann-Whitney U test, n = 7). (**c**) Response amplitudes normalized by the maximum amplitude in each area. A two-way ANOVA showed significant effects of stimulus type (p < 0.0001), cortical area type (p = 0.0161) and their interaction (p = 0.0004). The Mann-Whitney U test with Holm’s correction detected significant differences in the POR and ECT compared with the values in the V1 (**p < 0.01, n = 7).

To investigate the preference of stimulus size in each area, imaging was performed using moving discs with diameters between 0.15° and 10°, and response amplitudes were compared between the V1, POR and ECT using the same circular ROIs with a diameter of nine pixels or 0.3 mm. The three areas responded to a small moving disc with a diameter of 0.3° (Fig. 6a,b). The amplitudes of the V1 responses linearly increased as the disc diameter was enlarged from 0.3° to 10°. In contrast, response amplitudes in the POR and ECT were almost constant when the disc diameter was between 0.3° and 5°. A two-way ANOVA showed significant effects of stimulus type, cortical area type and their interaction. Response amplitudes elicited by the disc (diameter: 0.3°) were compared with those elicited by the discs in the range between 0.3° and 5° using the Mann-Whitney U test with Holm’s correction. No significant difference was found in the POR and ECT (Fig. 6b). We further normalized the response amplitudes by the maximum amplitude in each area (Fig. 6c). Using this method, we found significant differences between the POR/ECT and V1. These results indicate that the response-size relationship in the POR/ECT was clearly different from that in the V1. Under these experimental conditions, the amount of light emitted from the visual stimuli was proportional to the size of the moving discs. However, using black moving discs on a bright background, visual responses with similar size dependence were observed in the V1, POR and ECT (Fig. S4), indicating that the visual response amplitudes in the POR and ECT were not determined by disc size and independent of the brightness of the visual stimuli. These results are in agreement with the properties of activities in higher visual areas^22^ and behavioral studies suggesting the properties of higher visual areas^37^. These results seem to be in agreement with previous studies that responses of single neurons in higher order ventral stream areas of the rat become more independent on the luminance and contrast of the stimuli^22^ and that size tolerance of object recognition behavior is found in rats^37^.

**Figure 7:**
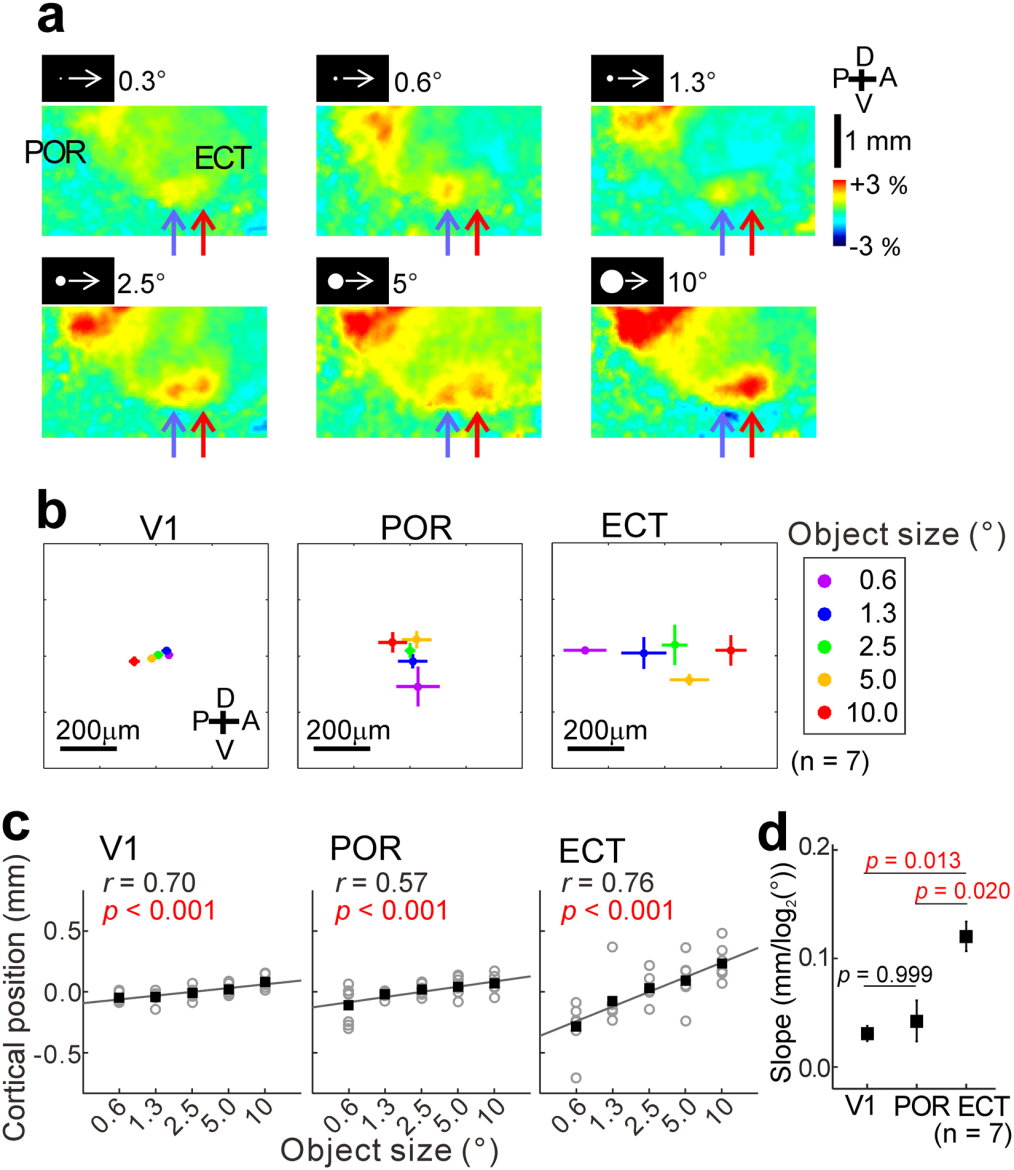
Cortical structure coding object size in the ECT. (**a**) Enlarged images of responses elicited by moving discs with various diameters. Violet and red arrows point to the response center in the ECT when the diameter was 0.3° and 10°, respectively. (**b**) Positional plots of maximal ΔF/F_0_ in the V1 (left), POR (middle left) and ECT (middle right) obtained using objects of various sizes (right). The mean of the coordinates of maximal ΔF/F_0_ position to each stimulus in each area was used as the origin of the coordinates to superimpose the data in seven mice. (**c**) Correlation between object size and cortical position of maximal ΔF/F_0_ in the V1 (left), POR (middle) and ECT (right). Open circles: data in seven mice; filled squares: mean. Cortical position (vertical axis) was measured along the approximate straight lines connecting the response areas. Statistical significance of correlation was estimated using the Spearman rank correlation test. (**d**) Comparison of slopes of approximate straight lines in (**c**) between the V1, POR and ECT. The Kruskal-Wallis test followed by the Dunn-Sidak test were used to estimate p values. Red values: p < 0.05 (n = 7).

Analysis of the ECT responses showed that the response amplitude was almost constant regardless of the stimulus size. However, the position of the response center was shifted from posterior to anterior parts of the ECT as the stimulus size was enlarged (Fig. 7a). In some mice, the ECT responses were observed in discreet patches responding specifically to small discs or large discs, as shown in Fig. 7a, while such discreet patches were not clearly observed in other mice. In the seven mice tested, we found a clear relation between stimulus size and the cortical position of the ECT responses (Fig. 7b). Cortical position of responses was shifted depending on object size in the V1, POR and ECT (Fig. 7c). We measured the slope of the cortical shift divided by stimulus diameter in a logarithmic scale for comparing the data in the three areas. The slope in the ECT was higher than that in the POR and much higher than in V1 (Fig. 7d). Similar results were obtained by using dark object stimuli (Fig. S5). Taken together, our results suggest that the ECT have some structures coding object size.

### Shape specificity in higher visual areas

**Figure 8:**
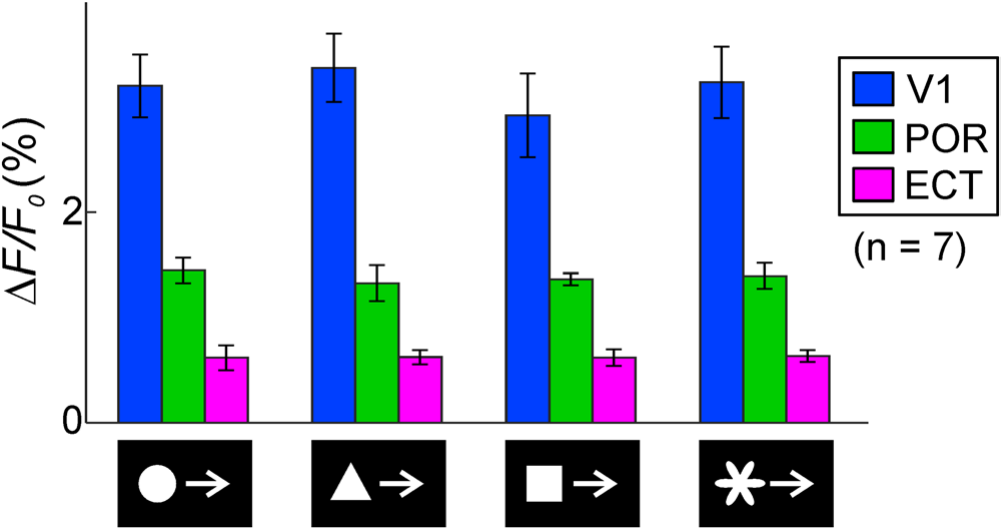
Responses to various shape stimuli. Response amplitudes to moving objects of various shapes in the V1 (blue), POR (green) and ECT (magenta). A two-way ANOVA showed no significant effects of stimulus type (p = 0.851) and of interaction with cortical area (p = 0.942), and a significant effect of cortical area type (p < 0.0001, n = 7).

In primates, one of the functions in the ventral pathway is shape recognition. Therefore, it is possible that neurons in the POR or ECT may have shape-specific properties. Unfortunately, this possibility is difficult to test using imaging techniques monitoring average neuronal activities. When various shapes including a disc were moved, responses in the V1, POR and ECT exhibited almost no preference to a particular shape (Fig. 8). In primates, it is widely known that there are cortical face areas where many neurons respond specifically to faces^9–12^. Therefore, we compared cortical responses to an upright moving face of a mouse, and compared the responses with those to a moving inverted face of a mouse. Although no clear difference in the responses to the different stimulus sets was found in the POR, the ECT in some mice responded better to an upright moving face (Fig. S6). Our results suggest that neuronal population in the POR and ECT can response to moving object stimuli regardless of their shape. However, this problem should be investigated by imaging methods at single neuronal level in future, because the present study does not exclude the possibility that mice might have shape-specific neurons in the ventral stream.

## Discussion

In the present study, we found that the POR and ECT in the mouse temporal cortex exhibited marked visual responses to moving objects. We used transgenic mice that express G-CaMP8^29^ in cortical excitatory neurons. The POR and ECT responded to moving objects in macroscopic cortical imaging and showed only broad retinotopic maps (Fig. 5). In contrast, some response patterns specific to the object size of the stimuli were observed in the ECT (Fig. 7). It has been reported that the V1 neurons project to the POR through the lateromedial area (LM) and laterointermediate area (LI), and that POR neurons project to the ECT^14^. The present study demonstrated that the V1, POR and ECT were sequentially activated by a moving disc in this order (Fig. 2). Comparison of the present results in mice and literature on the primates suggests that the POR and ECT are components of the mouse counterpart to the ventral stream in the primate visual system. However, there are some differences between the mouse system and the primate system.

The POR and ECT exhibited more vigorous responses to moving objects than to static objects (Fig. 1). In primates, however, it is known that the dorsal stream processes spatial and movement information of visual objects, while the ventral stream selectively responds to the texture or shape of visual objects^1–4^. Compared to the specific properties of the primate higher visual system, here, the POR and ECT of mice were more responsive to moving objects and not very sensitive to static objects. A previous electrophysiological study using rats has also reported that neurons in the temporal visual cortex are more responsive to moving stimuli than static stimuli^23^. Thus, the properties of the ventral stream in rodents and primates seem to be different.

Several factors may explain the species differences in the ventral stream. Firstly, it is conceivable that moving objects are more salient than static object, so that moving stimuli attract more attention in mice. It is well known that the neural responses to visual stimuli are enhanced when attention is directed to the stimuli^38^. In the present study, mice were not trained to perform any task, while primates are generally trained to perform a fixation task and pay attention to the stimulus monitor. If the mice used in the present study hardly attended to the stimulus monitor, it is no wonder that they paid more attention to moving objects depending on the saliency of the stimuli and that the visual responses to moving objects were thus enhanced.

The second potential explanation for the species differences in the ventral stream is that the entire visual system of mice may be innately more reactive to moving stimuli than the primate system, because mice are small animals and their movements are quick. Furthermore, their head inertia is small, and their head rotation speed is generally faster than that of primates. Therefore, moving objects appearing in the mouse visual field will also appear to be very fast. Since mice have only a small binocular region and their visual field is wider than that of primates, their visual system is likely specialized for quickly moving objects appearing in the wide visual field. The optimum speed of neurons in the MT field lies between 5°/s and 50°/s in primates^39^. In contrast, V1 axons projecting to the mouse dorsal stream have an optimum speed of up to 120°/s^16^. Therefore, it is likely that neurons in the ventral stream are also preferentially activated by moving stimuli in mice.

The third potential explanation for the species differences in the ventral stream is that, in mice, the identification of a visual object, which may be bait, another mouse, or a predator, may be critically dependent on the movement of the object. Even in primates, it has been reported that the dorsal and ventral streams are not completely independent and have mutual connections^40–42^. Therefore, specific visual responses to shape stimuli are observed not only in the ventral stream but also in the dorsal stream^43^. Conversely, visual responses to biological motion such as human-like movements are observed in the ventral stream^44^. It has also been reported that both the ventral and the dorsal streams contribute to shape perception^45^. Therefore, it is no wonder that visual responses in the mouse ventral stream are strongly affected by movement information of objects. The three potential explanations for the species differences in the ventral stream are not exclusive of each other, and further investigation of the species differences may give us some suggestions for understanding the evolutional development of the higher visual system in various species.

In the mouse ECT, the positional shift of visual responses was observed depending on the object size of the visual stimuli (Figs 7 and S5). These results may suggest that size invariance is not perfect in the ECT of mice. It is also possible that there are neurons selectively responding to a particular range of object size, and that these neurons are localized together in different cortical places of the ventral stream. This result appears to be contradictory to size-invariant response properties of neurons in the primate ventral stream^46,47^ and in the rat lateral extra striate cortex^22^. Recently, however, it has been reported that there are some areas selectively responding to the real-world size of visual objects in the human ventral stream^48^. A strawberry is small and a car is large in the real-world. This study thus suggests that strawberry-selective neurons may be located in specific areas responding to small objects while car-selective neurons may be found in other areas responding to large objects. Therefore, we speculate that the mouse ECT may have responded selectively to object size based on size-selectivity in the real world. For example, bait is usually smaller than a mouse, and predators are likely larger than a mouse. Although we used simple visual stimuli such as a disc, visual objects may be classified into different size categories in the mouse ECT. The separation of responsive areas in the ECT depending on object size suggests the presence of other areas selectively responding to a particular category of visual objects.

The primate ventral pathway is widely known to have shape-specific neurons, category-specific neurons and face-specific neurons^9–12,49^. We compared cortical responses to a moving upright face of a mouse, and compared the responses with those to a moving inverted face of a mouse. Although no clear difference in the responses to the different stimulus sets was found in the POR, the ECT in some mice responded better to an upright moving face (Fig. S6). Hopefully, more detailed investigations at the single-neuron level will reveal the whole picture of the what pathway in mice, and such studies, using various mouse strains, may generally help elucidating the mechanisms underlying visual cognitive functions in the near future.

## Materials and Methods

### Ethical approval and animals

The experimental procedures in the present study were approved by the committee for animal care at Niigata University (approval number: 372-7). All experiments were performed in accordance with approved guidelines and regulations. We used 32 male transgenic mice at postnatal months 1–4, obtained from crossing Emx1-Cre and G-CaMP8-flox (reporter) mice. The animals were housed in cages with *ad libitum* access to food and water, and were kept on a 12-h light/dark cycle. Mice received pet milk (MARUKAN Co., Ltd., Osaka, Japan) or 10% saccharose (Wako, Osaka, Japan) at habituation to handling, and at head fixation before and after imaging, to alleviate stress.

### Surgical operation required for imaging

Mice were anesthetized using isoflurane (Wako, Osaka, Japan). The concentration of isoflurane was 3–4% for induction and 1–2% during maintenance. The eyes were protected with ophthalmic ointment (Rinderon, Shionogi & Co., Ltd., Osaka, Japan), and temperature was maintained with a heating pad. After local anesthesia using bupivacaine (AstraZeneca, London, UK), the skin and right temporal muscle were incised. A metal head post was attached to the skull with dental cement (super bond, Sun Medical Co., Ltd., Shiga, Japan). The skull over the right visual cortex and temporal cortex was covered with clear dental cement and transparent nail polish (Seche Vite, Seche Ltd., CA, USA) to protect the surface of the skull. After surgery, mice were fed with a gel diet containing carprofen (MediGel CPF, Clear H_2_O, ME, USA) and small food pellets (CMF sprout, Oriental Yeast Co., Ltd., Tokyo, Japan) for a few days before the imaging experiments.

### Visual stimulation

Visual stimuli were generated by a program based on Matlab (The MathWorks, Inc., MA, USA) and the Psychtoolbox (http://www.psychtoolbox.org). Stimuli were presented on a gamma-corrected LCD monitor (FS2333, Eizo, Ishikawa, Japan), either placed at an angle of 45°or directly in front of the mice. The distance from the left eye was 30 cm. To avoid perturbation of fluorescence measurements using blue and green lights, the objective lens and imaging region was sealed from the light of the visual stimuli by using shading cloth and black tape. In preliminary experiments and those in Fig. 2, the surface of the monitor was further covered with a filter passing red light with λ > 600 nm (Sharp cut filter, Tokyo Butai Showmei Co., Ltd, Tokyo, Japan). Diameters of moving object stimuli ranged from 0.15° to 10°; speed ranged from 15°/s to 240°/s; and duration was 0.58–4.0 s. When rotationally moving discs were used for investigating speed preferences or retinotopic maps, rotating diameters were 38° (Figs 3 and 4) or 6° (other figures). Drifting sinusoidal gratings were also used in Fig. 1. The spatial frequency was 0.04 cycle/°, the temporal frequency was 2 Hz, direction was rightward. The diameter of patterns was 50° and the duration was 1.0 s. When static discs were used, the diameter was 5°. The brightness of all visual stimuli was <2 cd/m^2^.

### Auditory stimulation

Tones were generated by a program based on LabVIEW (National Instruments, Austin, TX, USA) at a sampling rate of 500 kHz. Sounds were low-pass filtered at 100 kHz. Pure tones at frequencies of 5 kHz and 30 kHz were amplitude-modulated by a 20-Hz sine wave. A speaker (SR-303, Stax, Saitama, Japan) was set at 35 cm in front of the mice. The sound intensity was 30–40 dB SPL using a sound level meter (Type 2610, Bruel & Kjær, Nærum, Denmark). The sound duration was 500 ms with a rise/fall time of 10 ms.

### Imaging experiments

Imaging was performed by using a MZ16F fluorescence microscope (Leica, Wetzlar, Germany) with a cooled CCD camera system (AQUACOSMOS with ORCA-R2 camera, Hamamatsu Photonics, Shizuoka, Japan) and an LED illumination system (UHP-Mic-LED-480, Prizmatix Ltd., Givat-Shmuel, Israel). Excitation was filtered at 450–490 nm and emission was filtered at 500–550 nm. A cortical region (5.6 × 4.3 mm or 168 × 128 pixels after binning) was imaged at a frame rate of 30 Hz (Figs 1 and 2, S1 and S2) or 10 Hz (all other figures). A dark frame was taken and subtracted from all acquired images in advance. The chronic imaging area was adjusted in reference to the cortical blood vessel images in imaging experiments using the same mice on separate days. Trials were repeated at 20-s intervals and averaged over 20 trials. Images were analyzed with a program based on Matlab. Spatial averaging of 5 × 5 pixels was applied. Time courses of signal intensity changes for each pixel were calculated as ΔF/F_0_, where ΔF is the increase in the fluorescence intensity and F_0_ is the average intensity during 300 ms, immediately before the stimulus onset. To remove the effect of light scatter, which is generated when emission light proceeds through the brain parenchyma, the Lucy-Richardson deconvolution was applied to ΔF/F_0_ images, where the width of a Gaussian was 0.2 mm^32,50^. The sample response images were obtained as temporally averaged images during 300 ms around the peak time.

### Data analysis

Retinotopic maps were produced from response images of rotating disc stimuli presented at multiple positions on a monitor in 29 animals. A retinotopic preference of each pixel in cortical images was defined as the peak of the Gaussian that fitted the response amplitude to each stimulus. Retinotopic maps were visualized by overlaying responses to stimuli presented at three retinotopic positions in different colors^19^. Borderlines of known visual areas were determined based on the retinotopic map^17,19^. To identify response peak pixels, V1 and POR areas were defined based on their response regions in response images of each animal. To identify a pixel of the peak maximum, the ECT area was defined as a rectangular area with 60 × 30 pixels located just anterior to a functional standard point in the V1 and just dorsal to the rhinal vein; the functional standard point in the V1 of each animal was determined as the peak response position to moving disc stimuli from left to right presented in front of the animal. The ECT area included ventral parts of the ectorhinal cortex and the temporal association cortex. An area size of the ECT was defined from the area where ΔF/F_0_ > 60% of maximal ΔF/F_0_. Regions posterior from the transverse sinus and lateral from the caudal rhinal vein were excluded from each area. Circular ROIs with a diameter of nine pixels or 0.3 mm were used for calculating response amplitudes. Response peaks in the V1, POR, and ECT areas were determined in the sample response images, and the ROIs were placed at the response peak in each area. Response amplitudes were calculated as temporal averages during 500 ms around the peak time in each ROI. When response positions in the cortex were analyzed, the mean of the coordinates of maximal ΔF/F_0_ positions to each stimulus in each area in each mouse was used as the origin of the coordinates to superimpose the data in multiple animals. Preferred speeds were defined as the peak of Gaussian that fitted to response amplitude of each stimulus speed.

### Statistical analysis

Response amplitudes in cortices to several visual stimuli were analyzed with a two-way ANOVA with area and stimulus type as factors. For multiple comparisons, the Kruskal-Wallis test followed by the Dunn-Sidak test, or the Mann-Whitney U test with Holm’s correction was used. Statistical significance of correlation was estimated using the Spearman rank correlation test. Error bars show SEM in all figures.

## Electronic supplementary material

Supplementary figures

## References

[1] Rizzolatti RG, Matelli M (2003). Two different streams form the dorsal visual system: anatomy and functions. Exp. Brain. Res..

[2] Goodale MA, Milner D (1992). Separate visual pathways for perception and action. Trends Neurosci..

[3] Ungerleider LG, Haxby JV (1994). “What” and “where” in the human brain. Curr. Opin. Neurobiol..

[4] Bell, A. H., Pasternak, T. & Ungerleider, L. G. Ventral and dorsal cortical processing streams. *The New Visual Neurosciences* (Eds Werner, J. S. & Chalupa, L. M.) 227–242 (The MIT Press: Cambridge, MA, 2013).

[5] Grill-Spector K (2006). High-resolution imaging reveals highly selective nonface clusters in the fusiform face area. Nat. Neurosci..

[6] Kiani R, Esteky H, Mirpour K, Tanaka K (2007). Object category structure in response patterns of neuronal population in monkey inferior temporal cortex. J. Neurophysiol..

[7] Kriegeskorte N (2008). Matching categorical object representations in inferior temporal cortex of man and monkey. Neuron.

[8] Peelen MV, Downing PE (2005). Selectivity for the human body in the fusiform gyrus. J. Neurophysiol..

[9] Tanaka K, Saito H, Fukada Y, Moriya M (1991). Coding visual images of objects in the inferotemporal cortex of the macaque monkey. J. Neurophysiol..

[10] Tsao YD, Freiwald WA, Tootell RBH, Livingstone MS (2006). A cortical region consisting entirely of face-selective cells. Science.

[11] Weiner KS, Grill-Spector K (2013). Neural representations of faces and limbs neighbor in human high-level visual cortex: evidence for a new organization principle. Psychol. Res..

[12] Rolls ET (2007). The representation of information about faces in the temporal and frontal lobes. Neuropsychologia.

[13] Wang Q, Gao E, Burkhalter A (2011). Gateways of ventral and dorsal streams in mouse visual cortex. J. Neurosci..

[14] Wang Q, Sporns O, Burkhalter A (2012). Network analysis of corticocortical connections reveals ventral and dorsal processing streams in mouse visual cortex. J. Neurosci..

[15] Andermann ML, Kerlin AM, Roumis DK, Glickfeld LL, Reid RC (2011). Functional specialization of mouse higher visual cortical areas. Neuron.

[16] Glickfeld LL, Andermann ML, Bonin V, Reid RC (2013). Cortico-cortical projections in mouse visual cortex are functionally target specific. Nat. Neurosci..

[17] Marshel JH, Garrett ME, Nauhaus I, Callaway EM (2011). Functional specialization of seven mouse visual cortical areas. Neuron.

[18] Matsui T, Ohki K (2013). Target dependence of orientation and direction selectivity of corticocortical projection neurons in the mouse V1. Front. Neural Circuits.

[19] Murakami T, Matsui T, Ohki K (2017). Functional segregation and development of mouse higher visual areas. J. Neurosci..

[20] Tohmi M, Meguro R, Tsukano H, Hishida R, Shibuki K (2014). The extrageniculate visual pathway generates distinct response properties in the higher visual areas of mice. Curr. Biol..

[21] Smith IT, Townsend LB, Huh R, Zhu H, Smith SL (2017). Stream-dependent development of higher visual cortical areas. Nat. Neurosci..

[22] Tafazoli S (2017). Emergence of transformation-tolerant representations of visual objects in rat lateral extrastriate cortex. Elife.

[23] Vermaercke B (2014). Functional specialization in rat occipital and temporal visual cortex. J. Neurophysiol..

[24] Shibuki, K., Hishida, R., Kitaura, H., Takahashi, K. & Tohmi, M. Coupling of brain function and metabolism: Endogenous flavoprotein fluorescence imaging of neural activities by local changes in energy metabolism. *Handbook of Neurochemistry and Molecular Neurobiology* (Eds Lajtha, A., Gibson, G. E. & Dienel G. A.) 321–342 (Springer: Boston, MA, 2007).

[25] Shibuki K (2003). Dynamic imaging of somatosensory cortical activity in the rat visualized by flavoprotein autofluorescence. J. Physiol..

[26] Haider B, Häusser M, Carandini M (2013). Inhibition dominates sensory responses in the awake cortex. Nature.

[27] Koldenkova VP, Nagai T (2013). Genetically encoded Ca^2+^ indicators: Properties and evaluation. Biochem. Biophys. Acta..

[28] Chen TW (2013). Ultrasensitive fluorescent proteins for imaging neuronal activity. Nature.

[29] Ohkura M (2012). Genetically encoded green fluorescent Ca^2+^ indicators with improved detectability for neuronal Ca^2+^ signals. Plos One.

[30] Wang Q, Burkhalter A (2007). Area map of mouse visual cortex. J. Comp. Neurol..

[31] Paxinos, G. & Franklin, K. B. J. *The Mouse Brain in Stereotaxic Coordinates*. (Academic Press: San Diego, CA, 2013).

[32] Tsukano H (2016). Quantitative map of multiple auditory cortical regions with a stereotaxic fine-scale atlas of the mouse brain. Sci. Rep..

[33] Polack PO, Contreras D (2012). Long-range parallel processing and local recurrent activity in the visual cortex of the mouse. J. Neurosci..

[34] Niell CM, Stryker MP (2008). Highly selective receptive fields in mouse visual cortex. J. Neurosci..

[35] Dumoulin SO, Wandell BA (2008). Population receptive field estimates in human visual cortex. Neuroimage.

[36] Tanaka K (1996). Inferotemporal cortex and object vision. Annu. Rev. Neurosci..

[37] Tafazoli S, Di Filippo A, Zoccolan D (2012). Transformation-tolerant object recognition in rats revealed by visual priming. J. Neurosci..

[38] Treue S (2001). Neural correlates of attention in primate visual cortex. Trends Neurosci..

[39] Maunsell JHR, Van Essen DC (1983). Functional properties of neurons in middle temporal visual area of the macaque monkey. I. Selectivity for stimulus direction, speed, and orientation. J. Neurophysiol..

[40] Nassi JJ, Callaway EM (2009). Parallel processing strategies of the primate visual system. Nat. Rev. Neurosci..

[41] Distler C, Boussaoud D, Desimone R, Ungerleider LG (1993). Cortical connections of inferior temporal area TEO in macaque monkeys. J. Comp. Neurol..

[42] Rosa MGP (2009). Connections of the dorsomedial visual area: Pathways for early integration of dorsal and ventral streams in extrastriate cortex. J. Neurosci..

[43] Sereno ME, Trinath T, Augath M, Logothetis NK (2002). Three-dimensional shape representation in monkey cortex. Neuron.

[44] Grossman ED, Blake R (2002). Brain areas active during visual perception of biological motion. Neuron.

[45] Zachariou V, Klatzky R, Behrmann M (2014). Ventral and dorsal visual stream contributions to the perception of object shape and object location. J. Cogn. Neurosci..

[46] Konen CS, Kastner S (2008). Two hierarchically organized neural systems for object information in human visual cortex. Nat. Neurosci..

[47] Sawamura H, Georgieva S, Vogels R, Vanduffel W, Orban GA (2005). Using functional magnetic resonance imaging to assess adaptation and size invariance of shape processing by humans and monkeys. J. Neurosci..

[48] Konkle T, Oliva A (2012). A real-world size organization of object responses in occipitotemporal cortex. Neuron.

[49] Gross CG (2008). Single neuron studies of inferior temporal cortex. Neuropsychologia.

[50] Issa JB (2014). Multiscale optical Ca^2+^ imaging of tonal organization in mouse auditory cortex. Neuron.

