# Supplementary material for: Higher visual responses in the temporal cortex of mice
Source: Sci Rep. 2018 Jul 24;8:11136. doi: 10.1038/s41598-018-29530-3 (PMC6057875; doi:10.1038/s41598-018-29530-3)
Supplement: Supplementary file 1 — Supplementary figures [file 41598_2018_29530_MOESM1_ESM.docx]

**SUPPLEMENTARY FIGURES**

**Higher visual responses in the temporal cortex of mice**

Nana Nishio^1^, Hiroaki Tsukano^1^, Ryuichi Hishida^1^, Manabu Abe^2^, Junichi Nakai^3, 4^, Meiko Kawamura^2^, Atsushi Aiba^5^, Kenji Sakimura^2^, Katsuei Shibuki^1^

1. Department of Neurophysiology, Brain Research Institute, Niigata University,

Niigata 951-8585, Japan

1. Department of Cellular Neurobiology, Brain Research Institute, Niigata University,

Niigata 951-8585, Japan

1. Graduate School of Science and Engineering, Saitama University,

Saitama, 338-8570, Japan

1. Brain and Body System Science Institute, Saitama University,

Saitama, 338-8570, Japan

1. Laboratory of Animal Resources, Center for Disease Biology and Integrative Medicine,　Faculty of Medicine, The University of Tokyo, Tokyo 113-0033, Japan

Correspondence: Katsuei Shibuki, Department of Neurophysiology, Brain Research Institute, Niigata University, 1-757 Asahi-machi, Chuo-ku, Niigata 951-8585, Japan

Phone: +81-25-227-0625; Fax: +81-25-227-0628; E-mail: shibuki@bri.niigata-u.ac.jp


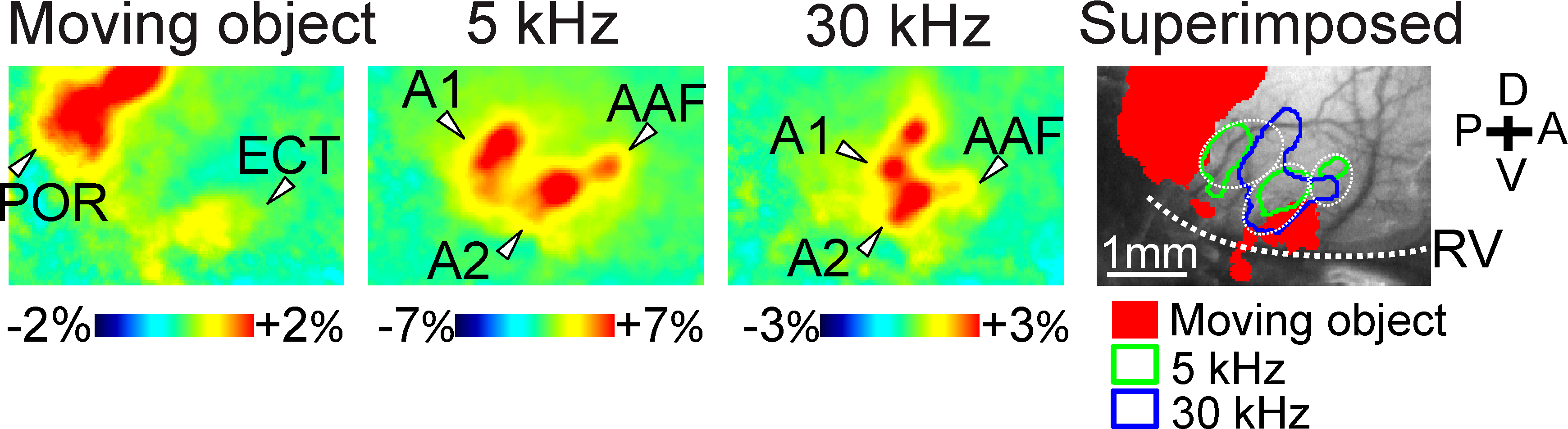


**Supplementary Figure S1. Relative positions of visual and auditory responses.**

Responses to a moving object (left), 5-kHz (middle left) or 30-kHz tonal stimuli (middle right) in the temporal cortex of the same mouse. Superimposed visual and auditory responses are also shown (right). The primary auditory cortex (A1), secondary auditory field (A2), and anterior auditory field (AAF) can be identified (fine dotted lines in the right panel). Thick dotted line shows the rhinal vein (RV).


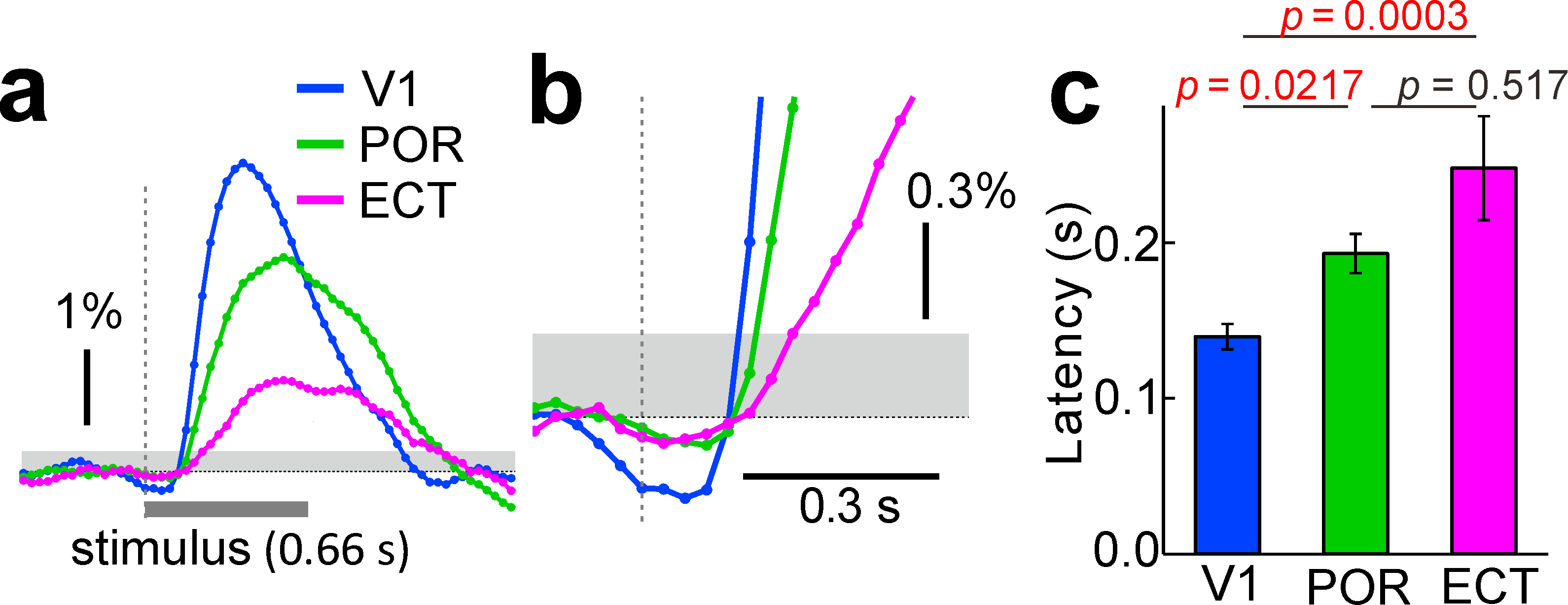


**Supplementary Figure S2. 2 SD latency of visual responses in the V1, POR and ECT.**

**a,** Fluorescence changes in the V1 (blue), POR (green) and ECT (magenta). ROIs are shown in Fig. 2b. Dashed line and dark gray bar indicate stimulus onset and stimulus presentation, respectively. Light gray area represents 2 SD range of the baseline. **b,** Enlarged traces shown in (**a**). **c,** 2 SD latency in the V1, POR and ECT. Statistical significance was evaluated by the Kruskal-Wallis test followed by the Dunn-Sidak test. Red values: p < 0.05.


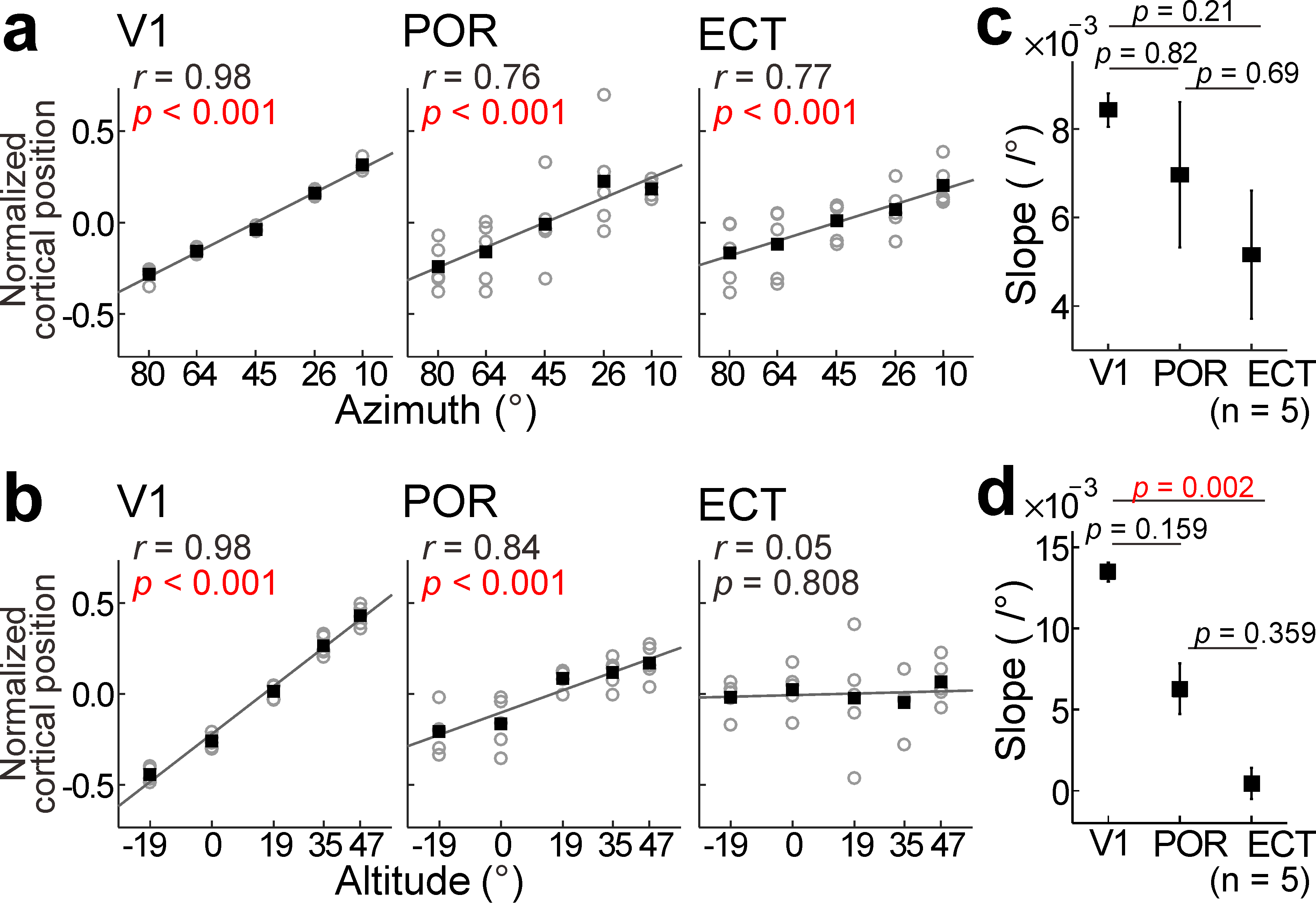


**Supplementary Figure S3. Quantitative analysis of normalized retinotopic structures.**

**a, b,** Correlation between stimulus positions in the horizontal (**a**) or vertical (**b**) visual field and normalized cortical positions of maximal ΔF/F_0_ in the V1 (left), POR (middle) and ECT (right). Open circles: data in five mice; filled squares: mean. Plots were obtained from the responses to the stimuli presented at 0° in altitude (**a**) or 27° in azimuth (**b**). Normalized cortical position (vertical axis) was observed along the approximate straight lines connecting the response peaks within each responsive area. Statistical significance of correlation was estimated using the Spearman rank correlation test. **c, d,** Comparison of slopes of approximate straight lines in (**a**, **b**) between the V1, POR and ECT. Statistical significance in five mice was evaluated by the Kruskal-Wallis test followed by the Dunn-Sidak test. Red values: p < 0.05.


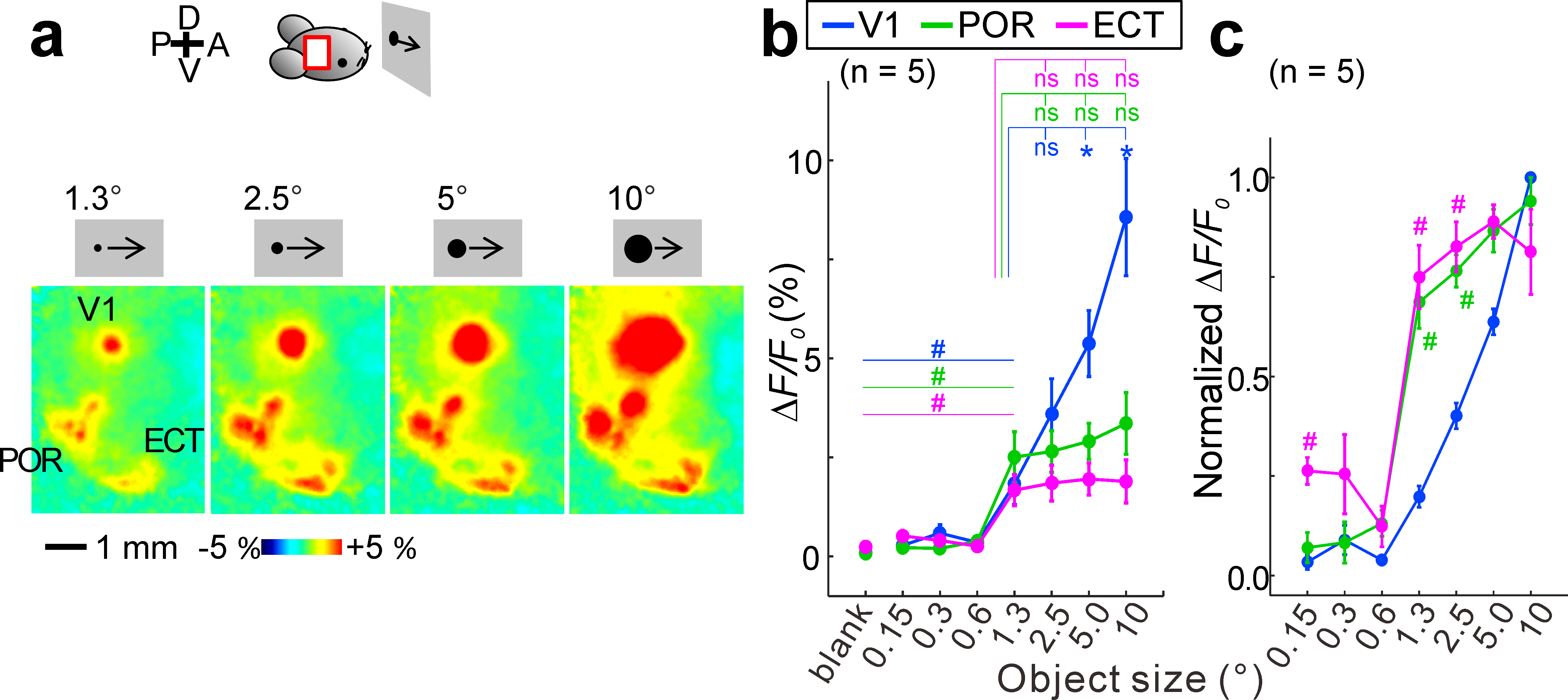


**Supplementary Figure S4. Response amplitudes to dark moving discs of various sizes.**

**a,** Responses to dark moving discs with a diameter between 1.3° and 10°. **b,** Response amplitudes and diameters of the dark moving discs. A two-way ANOVA showed significant effects of stimulus type, cortical area type and their interaction (p < 0.0001, each). Response amplitudes elicited by the stimulus with a diameter of 1.3°was significantly larger than the amplitudes with no stimulus (blank, # p < 0.01, the Mann-Whitney U test). Response amplitudes at 1.3° were not significantly different from those at 2.5-10° in the POR and ECT. However, significant differences were observed in the V1 (* p < 0.05, the Mann-Whitney U test). **c,** Response amplitudes normalized by the maximum amplitude of each cortex were shown. A two-way ANOVA showed significant effects of stimulus type, area type and their interaction (p < 0.0001, each). The Mann-Whitney U test detected significant differences (# p < 0.01) in the POR and ECT, when compared with the corresponding values in the V1.


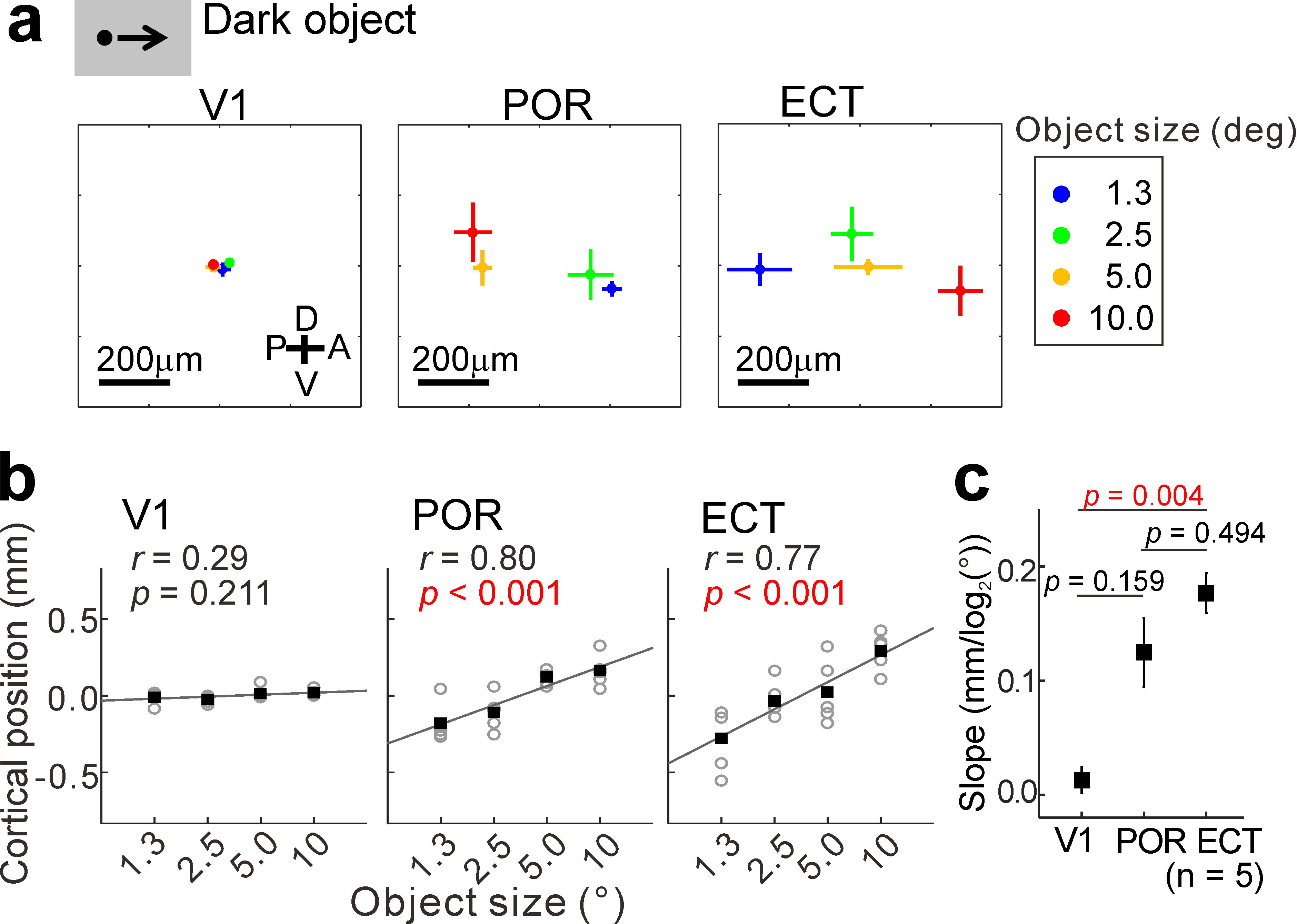


**Supplementary Figure S5. Cortical structures coding dark object size in the ECT.**

**a,** Cortical positional plots of maximal ΔF/F_0_ in the V1 (left), POR (middle) and ECT (middle) with dark objects at various sizes. The mean and SEM of the coordinates of maximal ΔF/F_0_ (n = 5) are shown. **b,** Correlation between object sizes and cortical positions of maximal ΔF/F_0_ in the V1 (left), POR (middle) and ECT (right). Open circles: data in five mice; filled squares: mean. Statistical significance of correlation was estimated using the Spearman rank correlation test. **c,** Slopes obtained from the approximate straight lines connecting the response areas were compared by the Kruskal-Wallis test followed by the Dunn-Sidak test between the V1, POR and ECT. Red values: p < 0.05.


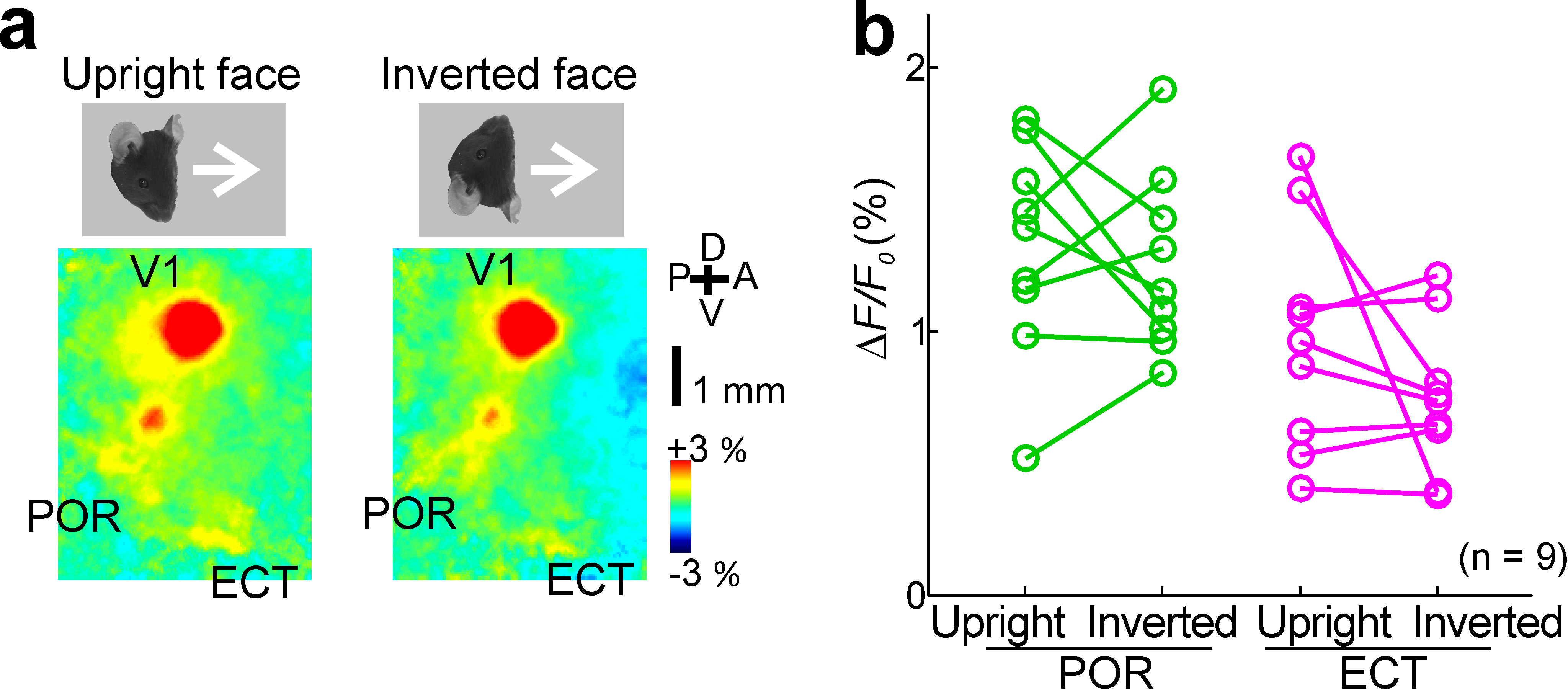


**Supplementary Figure S6. Responses to moving upright or inverted face of a mouse in the POR and ECT.**

**a,** Visual responses to moving upright or inverted face stimulation. **b,** Amplitudes of responses to upright or inverted face stimulation in the POR and ECT.
